# Lab partners: oocytes, embryos and company. A personal view on aspects of oocyte maturation and the development of monozygotic twins

**DOI:** 10.1590/1984-3143-AR2023-0049

**Published:** 2023-07-24

**Authors:** Burkhard Meinecke, Sabine Meinecke-Tillmann

**Affiliations:** 1 Institut für Reproduktionsbiologie, Tierärztliche Hochschule Hannover, Hanover, Germany; 2 Ambulatorische und Geburtshilfliche Veterinärklinik, Justus-Liebig-Universität Giessen, Giessen, Germany; 3 Institut für Tierzucht und Haustiergenetik, Justus-Liebig-Universität Giessen, Giessen, Germany

**Keywords:** oocyte maturation, soma-germ interactions, monozygotic twins, demi-embryos, maternal effects

## Abstract

The present review addresses the oocyte and the preimplantation embryo, and is intended to highlight the underlying principle of the “nature versus/and nurture” question. Given the diversity in mammalian oocyte maturation, this review will not be comprehensive but instead will focus on the porcine oocyte. Historically, oogenesis was seen as the development of a passive cell nursed and determined by its somatic compartment. Currently, the advanced analysis of the cross-talk between the maternal environment and the oocyte shows a more balanced relationship: Granulosa cells nurse the oocyte, whereas the latter secretes diffusible factors that regulate proliferation and differentiation of the granulosa cells. Signal molecules of the granulosa cells either prevent the precocious initiation of meiotic maturation or enable oocyte maturation following hormonal stimulation. A similar question emerges in research on monozygotic twins or multiples: In Greek and medieval times, twins were not seen as the result of the common course of nature but were classified as faults. This seems still valid today for the rare and until now mainly unknown genesis of facultative monozygotic twins in mammals. Monozygotic twins are unique subjects for studies of the conceptus-maternal dialogue, the intra-pair similarity and dissimilarity, and the elucidation of the interplay between nature and nurture. In the course of in vivo collections of preimplantation sheep embryos and experiments on embryo splitting and other microsurgical interventions we recorded observations on double blastocysts within a single zona pellucida, double inner cell masses in zona-enclosed blastocysts and double germinal discs in elongating embryos. On the basis of these observations we add some pieces to the puzzle of the post-zygotic genesis of monozygotic twins and on maternal influences on the developing conceptus.

## Introduction

“Knowledge of history allows the conquest of the future. This is especially true for embryo transfer with all its ramifications, which has become one of the most challenging frontiers in theriogenology” (Jöchle, 1983, p. 293). About 50 years ago, physiological aspects of oocytes and embryos came into research focus, mainly driven by questions on bovine embryo transfer (ET), which enhanced the interest in reproductive biology in farm animals. Depending on the species, each year thousands of embryos are produced in vitro or collected in vivo and transferred to final recipients. Basis of these events is the physiological and undisturbed maturation of fertilizable oocytes. Resulting preimplantation embryos, regardless of their in vivo or in vitro origin, show tremendous regulatory capacities, and in some cases more than half of the blastomeres can be lost before further development completely fails. The ability to compensate for a reduced blastomere count is the basis of facultative or induced polyembryonic development. Artificial splitting of high quality embryos and single transfer of the resulting halves allows, in dependence on the manual skills of the operator, a 30% to 40% greater embryo transfer success in cattle compared to the transfer of intact embryos.

Despite the successful implementation of the newly developed reproductive technologies into animal production and human reproductive medicine, essential questions remained only incompletely answered. Two of the most puzzling processes, the preovulatory maturation of the oocyte and the origin of monozygotic twins, accompanied our research and are covered by the following sections of the review.

## Oocyte maturation: the perspective of the pig

### Animal models

Experiments of Pincus and Enzmann (1935) and of Edwards (1965) initiated a countless number of studies about in vitro maturation (IVM) of mammalian oocytes. Many of these studies were conducted to improve the quality of IVM-oocytes for use in animal biotechnology but also to decipher basic mechanisms of mammalian oocyte maturation. Female meiosis is an outstanding model for studying the cell cycle since oocytes become arrested in prophase1 (equivalent to the G2-phase of mitotic cells) during fetal development until just before ovulation when they proceed to metaphase 2 (M2). Newly developed methods have led to a significant broadening and deepening of knowledge about the regulation of oogenesis. It became evident that key molecules of this complex process appeared rather early in vertebrate evolution (Dalbies-Tran et al., 2020). At the same time, these new findings also shed light on special features of the animal species and the necessity to verify observations in each of them. The ability to manipulate the mouse genome makes mice the premier model organism for genetic approaches to study the molecular mechanisms underlying the maturation of oocytes. However, different adaptations of physiological regulatory circuits make it necessary to develop new model systems according to the respective questions. For example, in contrast to mice, pig oocytes depend on mRNA- and protein synthesis for germinal vesicle breakdown (GVBD) and show a chromosome dependent spindel formation (Fulka et al., 1986; Meinecke and Meinecke-Tillmann, 1993; Miyano et al., 2007). The following review about some aspects of oocyte maturation focuses on the pig. Additional information derived from other species will be used in order to complete the description of the relevant processes.

### Physiological considerations

Porcine follicular development from the primordial to the preovulatory stage lasts about 120 days (Hunter, 2000). In the course of follicular maturation it is inevitable that the countless factors influencing this evolvement cause a considerable heterogeneity of follicles and their oocytes. (Moor and Dai, 2001). However, in vivo a cohort of heterogeneous oocytes is being transformed to a selected few of widely homogenous oocytes before ovulation occurs. Maturation of pig oocytes is initiated by the preovulatory endogenous LH surge at the end of a follicular phase of 4 to 6 days. Recruitment from the pool of about 100 developing follicles occurs after the pulsatile GnRH-LH secretion changed from a lesser frequency/greater amplitude to a greater frequency/lesser amplitude pattern. In pigs FSH is important for increasing the number of follicles that reach the medium/larger sized category, whereas LH is necessary for the further growth of these follicles to preovulatory size. Luteinizing hormone (LH) stimulates follicular estrogen synthesis in theca interna and granulosa cells which have expressed sufficient LH receptors. Two to 3 days before ovulation, LH pulsatility and FSH secretion decrease to hardly detectable levels, while estrogen concentrations reach their maximum. On the first day of standing estrus (Day 0 of the estrus cycle) and on Day 1 estrogens dominate follicular steroid hormone synthesis and output. About 12 h before ovulation occurs on Day 2, estrogen synthesis has ceased and follicular levels have dropped to a tenth of their original maximum levels (Eiler and Nalbandov, 1977; Meinecke et al., 1987; Soede et al., 2011; Knox, 2019). Ovulations take place 44 h ± 3 h after the onset of the LH surge and last about 3 h (Soede et al., 1994). Compared to adult sows, induction of ovulation in prepubertal gilts with an equine chorionic gonadotropin/human chorionic gonadotropin (eCG/hCG) regimen is accompanied by an altered pattern of follicular growth and steroid hormone synthesis, as well as a greater variability in oocyte maturation, and a prolonged ovulation process (Ainsworth et al., 1980; Meinecke et al., 1984; Foxcroft and Hunter, 1985; Wiesak et al., 1990; Soede et al., 1998). The considerable aberration of morphological and biochemical parameters is also reflected in reduced developmental competence of oocytes from prepubertal gilts (Pinkert et al., 1989; Bagg et al., 2004, 2007).

### Nuclear changes

Nuclear changes in follicular oocytes at various times after the onset of estrus were reported by Spalding et al. (1955), or after hCG application by Hunter and Polge (1966). A first report on IVM of pig follicular oocytes revealed a coinciding time line between in vitro and in vivo nuclear maturation (Edwards, 1965). However, it soon became evident that the presence of a M2 figure is in no sense an adequate indication of normal maturation of the oocyte and that complete maturation comprises two separate but interacting entities (Leman and Dziuk, 1971; Motlík and Fulka, 1976; Mattioli et al., 1989). It turned out that in pigs, the transfer of IVM oocytes as well as of oocytes recovered from atretic follicles into previously mated recipients revealed a reduced and retarded cleavage rate of IVM oocytes. In contrast, the sperm penetration rate of oocytes from atretic follicles was equivalent to IVM oocytes, indicating that under in vivo conditions spermatozoa do not discriminate between in vitro matured, atretic, and ovulated oocytes (Meinecke and Meinecke-Tillmann, 1978a, b).

### Aquisition of meiotic competence

Although oocytes of follicles ≤ 1 mm in diameter are able to resume meiosis in vitro, only those of > 2 mm in diameter can complete the first meiotic division indicating a close relationship between follicular growth and oocyte maturation. During pig folliculogenesis, the ability to undergo GVBD and to proceed to metaphase 1 (M1) is acquired earlier than the ability to reach M2 (Tsafriri and Channing, 1975; Motlik et al., 1984). The competence to transit through post-GVBD stages undisturbed is only achieved following transcriptional silencing (McGaughey et al., 1979; Crozet et al., 1981; Motlik et al., 1984; Motlík and Fulka, 1986; Meinecke and Meinecke-Tillmann, 1998; Bjerregaard et al., 2004; Pan et al., 2018). Subsequent to removal of the nucleolus, fully grown porcine oocytes resume meiosis and proceed undisturbed to M2. Removal of the nucleolus induces GVBD in growing oocytes which normally are unable to resume meiosis, and the cell cycle proceeds to M2 (Fulka et al., 2003). These observations challenge the concept of the nucleolus as a mere ribosome factory and indicate an active role in preventing GVBD in growing oocytes. The changes in transcriptional activity are reflected by chromatin appearance and can be used to differentiate transcriptionally active oocytes from transcriptionally silent ones (Motlík and Fulka, 1986). The chromatin in porcine oocyte nuclei is initially decondensed in a non-surrounded-nucleolus (NSN) configuration, but subsequently condensed, forming a surrounded nucleolus (NS) configuration with a heterochromatin rim around the nucleolus (Sun et al., 2004). In the meantime, the morphological criteria have been further refined (Guthrie and Garrett, 2000; Sun et al., 2016; Pan et al., 2018; Lee et al., 2019). Although growing (≤ 90 μm in diameter) and fully grown pig oocytes (≥ 115 μm) contain comparable amounts of the two subunits of the maturation promoting factor (MPF), they are not able to activate the MPF to a sufficient extent. One of the causes of this incompetence is the continuous activation of a MPF-inhibiting kinase (WEE1B) which became phosphorylated by a persistent activity of cAMP-dependent protein kinase (PKA) (Christmann et al., 1994; Nishimura et al., 2009, 2012). Furthermore, growing oocytes are unable to establish an intact mitogen-activated protein kinase-pathway (MAPK3/1) required for full meiotic competence (Kanayama et al., 2002). Extracellular signal-regulated kinases (ERK1/2) are involved in early porcine folliculogenesis as evidenced by the marked intensity of activated MAPK3/1 immunolabeling in the cytoplasm of oocytes from primordial/primary, secondary and tertiary follicles (Moreira et al., 2013). Furthermore, Sun et al. (2016) highlighted the signaling pathways of MAPK3/1 required for the transition of chromatin from a decondensed to a condensed condition in growing pig oocytes.

### Prophase1 arrest

Since prophase1 arrest of mammalian oocytes could be maintained in vitro by a membrane-permeate form of cAMP or a cAMP phosphodiesterase inhibitor, early ideas of prophase1 arrest were based on the concept that premature resumption of meiosis is prevented either by cAMP which is produced by follicle cells and diffuses into the oocyte or by cAMP synthesized by the oocyte itself (Jaffe and Egbert, 2017). This concept held until the demonstration of a constitutive active G-protein-coupled-receptor 3 (GPR3), stimulating adenylyl cyclase in mice oocytes. The resulting elevated cAMP levels in the oocyte prevent resumption of meiosis (Kalinowski et al., 2004; Mehlmann et al., 2004). Creation of mouse oocytes defective in synthesis and degradation of cAMP has shown that both, GPR3 and phosphodiesterase 3A, are the most important regulators of intra-oocyte cAMP concentrations necessary to block resumption of meiosis (Vaccari et al., 2008; Norris et al., 2009). Phosphodiesterase 3A is competitively inhibited by cyclic guanosine monophosphate (cGMP) which is provided by granulosa cells and diffuses through gap junctions into the oocyte. Granulosa cell production of cGMP is controlled by natriuretic peptide stimulation of natriuretic peptide receptor 2 (NPR2) which is coupled to a guanylyl cyclase (Zhang et al., 2010). Convincing evidence of cAMP production by the oocyte itself, and the meiosis-inhibiting effect of cAMP have also been demonstrated in the pig (Rice and McGaughey, 1981; Mattioli et al., 1994). Likewise, proof has been given of the presence of the GPR3-adenylyl cyclase-cAMP system and cGMP inhibition of phosphodiesterase 3 A in porcine oocytes (Laforest et al., 2005; Sasseville et al., 2006; Morikawa et al., 2007; Yang et al., 2012; Zhang et al., 2012). Pig granulosa cells produce and secrete both B-type brain natriuretic peptide (BNP) as well as C-type natriuretic peptide (CNP), and are endowed with NPR1 and NPR2. Moreover, meiotic resumption of porcine oocytes has been inhibited by CNP signaling (Kim et al., 1992; Ivanova et al., 2003; Hiradate et al., 2014; Santiquet et al., 2014; Zhang et al., 2015).

The combined data suggest that subsequent to binding of CNP and BNP to NPR2, production of cGMP in porcine granulosa cells is activated which then passes through gap junctions into the oocyte. Prophase1 is maintained by high cGMP concentrations which competitively inhibit phosphodiesterase 3, thus retaining high intra-oocyte cAMP levels. The cAMP activates PKA which holds MPF in an inactive state by phosphorylating WEE1 (Shimaoka et al., 2009; Jaffe and Egbert, 2017). The cooperations between somatic and germinative compartments remain stable until increasing and persistent estrogen blood concentrations support the LH surge.

### Resumption of meiosis by LH signaling

Gonadotropin receptor expression of porcine ovarian follicles during the estrous cycle depends on both follicular development and stage of the estrous cycle. In small and medium ovarian follicles of prepubertal and adult pigs, follicle stimulating hormone receptors (FSHR) are highly expressed in granulosa cells and decline in mature follicles at estrus. In contrast to the luteinizing hormone receptor (LHR), the number of FSHR per granulosa cell and their binding affinity do not increase in the course of follicular development (Nakano et al., 1977). Luteinizing hormone receptor (LHR) expression in granulosa cells weakly starts in medium sized follicles and is increased in large follicles on Day 0 (Nakano et al., 1977; Daguet, 1979; Liu et al., 1998). Porcine cumulus cells express low numbers of LHRs and show no cAMP response upon LH stimulation (Mattioli et al., 1994). In vitro LHR-expression of porcine cumulus cells increases only after additional FSH stimulation (Shimada et al., 2003; Ozawa et al., 2008). Simultaneously with the follicular development, the adenylyl cyclase-system of porcine granulosa cells reacts increasingly responsive to hCG. Following LH stimulation of follicles (6-10 mm in diameter) about 7.000 molecules of cAMP are formed/sec/granulosa cell (Lee, 1976). Since follicles of that size class contain about 2 to 3 million granulosa cells (Foxcroft and Hunter, 1985) one can imagine that follicles become submerged by cAMP molecules. Moreover, LH signaling is extended by internalization of the receptors after LH binding, such that signaling may continue from endosome compartments even in the absence of extracellular LH (Johnson and Jonas, 2020). Prophase1 of oocytes remains arrested when pig ovarian follicles are isolated and subsequently cultivated without gonadotropins, whereas addition of LH/FSH or eCG/hCG to the culure medium induces resumption of meiosis (Gérard et al., 1979; Meinecke and Meinecke-Tillmann, 1981). The LHR is a G protein-coupled receptor and, upon binding of its ligand, the activation of cAMP-dependent targets is stimulated (Mattioli and Barboni, 2000; Choi and Smitz, 2014). Furthermore, signal transduction also takes place via additional G protein-independent pathways (Johnson and Jonas, 2020). However, the primary targets of the LH signal, initiating the ovulation process and the resumption of meiosis are a.) the steroidogenesis of the preovulatory follicle, b.) the gap junctional communication between follicle cells and the oocyte, c.) the natriuretic peptide system, and d.) the epidermal growth factor network.

- Steroidogenesis of the preovulatory follicle

In early studies, porcine follicles were cultivated in vitro after ovariectomy of eCG/hCG treated prepubertal gilts. It was shown that stimulation of progesterone secretion already started in follicles removed 15 min after intravenous hCG application, whereas resumption of meiosis was induced in follicles removed after at least 4 h following hCG (Meinecke and Meinecke-Tillmann, 1979). The time window for in vivo LH signaling targeting oocyte maturation corresponds with the results obtained by IVM of porcine cumulus oocyte complexes (Ebeling et al., 2007; Sasseville et al., 2009). In further investigations the relative amount of progestagens, androgens, and estrogens in follicular fluid and in follicular wall samples, revealed the change from estrogen to progesterone synthesis of the preovulatory follicle (Eiler and Nalbandov, 1977; Ainsworth et al., 1980; Meinecke, 1981; Foxcroft and Hunter, 1985; Meinecke et al., 1987). The role of steroid hormones in oocyte maturation has been debated for decades (Tsafriri and Motola, 2007) but recently became a subject of interest again. Estrogen promotes the natriuretic peptide driven production of cGMP in mouse granulosa cells and in cumulus cells, thus assisting meiotic arrest (Liu et al., 2017), whereas progesterone signaling via its receptor is essential for the resumption of meiosis and cumulus expansion in pigs (Yamashita et al., 2010). Our own investigations have demonstrated that an inhibition of MAPK3/1 by U0126 in porcine cumulus cells during gonadotropin induced IVM resulted in a cessation of progesterone synthesis by suppression of 3ß-hsd gene expression and an increase of estradiol synthesis by stimulating Cyp 19 a1 gene expression (Ebeling et al., 2011). The same effect had been noticed in cultured granulosa cells and cumulus oocyte complexes (COCs) of mice (Su et al., 2006). Thus, it can be assumed that the LH signal induces a differential expression of genes essential for estrogen and progesterone synthesis in cumulus cells as well as in granulosa cells and that this process is mediated by a MAPK3/1-dependent signaling pathway.

- Gap junctional communication between follicular cells and the oocyte

Pigs strongly express connexin 43 in cumulus cells, connexin 60 in oocytes, and connexin 45 in both oocytes and cumulus cells (Santiquet et al., 2013). Following hCG application to eCG pretreated pigs, coupling of cumulus cells and oocytes as determined by [3H]uridin uptake remains unchanged until about 32 h. At this point of time pig oocytes reach M1 and exhibit complete cumulus cell expansion (Motlik et al., 1986; Mattioli et al., 1988). Gonadotropin treatment (FSH/LH; eCG/hCG) causes a rise in the amount of connexin 43 protein in pig COCs corresponding to an increase in gap junctional communication during the initial phase of IVM, whereas GVBD is accompanied by closure of gap junctions (Shimada et al., 2001; Sasseville et al., 2009). During the first few hours of IVM the presence or absence of gonadotropins has hardly any effect on the gap junction network between porcine cumulus cells. Currently, gap junctional communication during porcine IVM is explained in terms of increasing and decreasing the total number of connexin 43 molecules in cumulus cells, thus varying the number of gap junctions and hence the gap junctional communication flow rate (Santiquet et al., 2013). However, it should be stressed that the capacity of cumulus cells to promote resumption and completion of oocyte meiosis is gonadotropin-dependent and that a rapid and profound decrease of gap junctional communication accompanies GVBD in porcine oocytes (Sasseville et al., 2009; Santiquet et al., 2012).

- The natriuretic peptide system

In rodent follicles, LH inhibits gualylyl xyclase activity of NPR2 via dephosphorylation by a rapid still unknown way, thus lowering the cGMP levels in the somatic compartment (Robinson et al., 2012; Egbert et al., 2014; Shuhaibar et al., 2015). Additionally, the LH-cAMP-PKA system of granulosa cells activates phophodiesterase 5 which also contributes to a reduction of somatic cGMP concentrations via hydrolytic cleavage (Egbert et al., 2016).

In pigs, the application of hCG to eCG pretreated animals decreased CNP as well as BNP concentrations (~ 80%) in follicular fluid at 18 h and 36 h after treatment, respectively. These observations indicate that under physiological conditions BNP and CNP jointly contribute to meiotic arrest, and LH attenuates this inhibitory effect by decreasing the expression levels of BNP and CNP in vivo (Hiradate et al., 2014; Zhang et al., 2015). The timely upregulation of the principal phosphodiesterase 3 activity in porcine cumulus cells requires FSH signaling (Sasseville et al., 2009), and so both, the CNP-NPR2 down-regulation and the upregulation of phosphodiesterase 3 activity lowers the cGMP concentrations in the somatic compartment. As a consequence, cGMP diffuses out of the oocyte down its concentration gradient. Consequently, the competitive inhibition of phosphodiesterase 3 by high cGMP levels in the oocyte is released and hydrolysis of cAMP begins (Jaffe and Egbert, 2017).

- The epidermal growth factor network

Activation of the EGF receptor after the LH surge is mediated by amphiregulin and epiregulin (Park et al., 2004). Synthesis of both peptides increases after LH stimulation in porcine granulosa cells, and after EGF receptor activation cGMP levels decrease in granulosa cells and in cumulus cells (Zhang et al., 2014). It is suggested that amphiregulin and epiregulin are released from granulosa cells into the extracellular space and diffuse to cumulus cells, where they lower cGMP concentrations. In addition, LH related EGF receptor signaling induces MAPK3/1 activation, mucification of the cumulus matrix, gap junction closure, and oocyte meiotic resumption in several mammals including the pig (Liang et al., 2005; Prochazka and Blaha, 2015). During porcine folliculogenesis, responsiveness to EGF signaling develops concomitantly with follicular growth (Marchal et al., 2001; Procházka et al., 2000). Porcine cumulus cells from small (<4 mm in diameter) and large (>4mm in diameter) follicles contain similar quantities of EGF receptor protein but following EGF stimulation only expanding cumulus cells from large follicles contain EGF receptors capable to activate intrinsic tyrosine phosphorylation (Prochazka et al., 2003). In addition, porcine COCs from small follicles expressed equivalent amounts of EGF receptor mRNA compared to COCs from large follicles. However, the former had less total EGF receptor protein, resulting in failed activation of phospho-EGF receptor and phospho-ERK1/2, despite of equivalent total ERK1/2 protein levels (Ritter et al., 2015). This underlines the importance of an intact EGF receptor signaling pathway, since MAPK3/1 in particular is of importance for cumulus expansion, resumption of meiosis, and ovulation.

### Germinal vesicle breakdown (GVBD)

The MPF complex consists of two components, a catalytic subunit, namely the cyclin dependent kinase 1 (CDK1), and a regulatory subunit, cyclin B (Dunphy and Newport, 1988; Nurse, 1990). Phosphorylation on threonine 161 by a Cdk activating kinase (CAK1) and dephosphorylation on Thr 14 and thyrosine 15 by the cell division phosphatase 25 (CDC25) activates CDK1 (Krek and Nigg, 1992; Solomon et al., 1992). The necessity of threonine 161 phosphorylation of CDK1 for MPF activation at meiotic resumption of porcine oocytes has been confirmed (Fujii et al., 2011). In immature porcine oocytes, high cAMP levels activate WEE1B which subsequently inactivates CDK1 (Nishimura et al., 2009). After in vivo/in vitro induction of meiotic resumption, declining cAMP levels in the oocyte inactivate WEE1B, followed by activation of CDC25 and the conversion of pre-MPF to MPF. It is suggested that the MPF activity during this early period is not sufficient to induce meiotic resumption because of still low cyclin B concentrations. The moderate MPF activity starts the cyclin synthesis which results in a further MPF activation and provokes GVBD (Shimaoka et al., 2009). Activation of MPF during IVM of pig oocytes occurs in close correspondence to GVBD but is influenced among other factors by media composition and quality of the oocytes (Naito and Toyoda, 1991; Wehrend and Meinecke, 2001; Setiadi et al., 2009). Since inhibition of protein synthesis as well as suppression of CDK1 activation prevents porcine GVBD, it was assumed that cyclin B synthesis is required for GVBD (Naito et al., 1995; Kubelka et al., 2002). However, the trigger of GVBD in pig oocytes might not be the cyclin synthesis but the dephosphorylation of pre-MPF. When porcine oocytes were injected with antisense cyclin RNAs (B1 and B2), they gradually underwent GVBD in the absence of cyclin B synthesis. Despite the resulting low MPF activity in these oocytes, they were able to activate a small amount of pre-MPF to induce GVBD, although the time line was retarded. This suggests that pig oocytes do not require cyclin synthesis for GVBD induction per se but they need either cyclin B1 or B2 synthesis for GVBD in a correct time course (Kuroda et al., 2004).

During IVM, the activities of MPF and MAPK3/1 increase around the time of GVBD. Maturation promoting factor exhibits two maxima at M1 and M2 with a temporary drop during M1 to M2-transition, whereas MAPK3/1 activities remain stable at peak levels until M2 (Mattioli et al., 1991; Inoue et al., 1995; Wehrend and Meinecke, 2001; Ye et al., 2003). In COCs connected to a piece of the mural granulosa cell layer, spontaneous maturation is prevented unless a combination of LH/FSH is present in the medium (Motlik et al., 1991; Ebeling et al., 2007). This in vitro model allows for a distinction between spontaneous and gonadotropin induced resumption of meiosis. In cumulus cells, FSH/LH induces an early and rapid U0126-insensitive MAPK3/1 phosphorylation, while U0126-susceptible MAPK3/1 phosphorylation occurs in the oocyte itself at GVBD (Ebeling et al., 2007). Since chromosome condensation can occur in the absence of MPF-activity, and GVBD can take place without MAPK3/1 activation, the specific roles of MPF and of MAPK3/1 in the oocyte have yet not fully been elucidated (Kubelka et al., 2002; Ye et al., 2003; Prochazka and Blaha, 2015; Kalous et al., 2018).

### Cytoplasmic maturation

Cytoplasmic maturation is an ill-defined process providing the oocyte with the ability to navigate through fertilization and early embryonic cleavage until completion of zygotic genome activation. Previous experiments have demonstrated that follicle size affects the competence of the oocyte to develop to the blastocyst stage (Marchal et al., 2002; Bagg et al., 2007). Induction of cytoplasmic maturation requires signal exchange between somatic cells and the oocyte as shown by the first successful IVF of porcine in vitro matured oocytes (Mattioli et al., 1989).

At present the efficiency of porcine in vitro production (IVP) of embryos is very low. Despite the many improvements of maturation and fertilization of the oocyte as well as cultivation of the early embryo under in vitro conditions, it has yet not been possible to achieve a significant enhancement in the overall process (Grupen, 2014; Chen et al., 2021). Involvement of the MAPK3/1 in cytoplasmic maturation had been demonstrated by our group through the role of the kinase during the oocyte aging process. By prolonging the culture period of porcine COCs from 46 h up to 72 h to induce oocyte aging, a significant decrease of the MAPK3/1 activity occurred during the first 12 h of aging and stabilized during a further prolonged culture time (Ebeling et al., 2010). Prematurely decreasing MAPK3/1 activities in aged MII porcine oocytes seem to hamper subsequent early embryonic development. Furthermore, a proportion of oocytes with abnormal anaphase II significantly increased after parthenogenetic activation of aged oocytes (Ma et al., 2005). However, our attempts to use MPF/MAPK3/1 monitoring as an indicator of cytoplasmic maturation during porcine IVM showed that this method is not suitable (Setiadi et al., 2009).

### Conclusion

Maturation into a developmentally competent oocyte under in vitro conditions seems to be particularly difficult in swine as compared to cattle. Despite the tremendous efforts during the past decades only limited success has been achieved. Main problems like heterogeneity of oocytes, polyspermic penetration, and aberrant early embryonic development, to name the most obvious examples, have yet not been fully understood. In almost all studies, oocytes from slaughtered prepubertal animals are used, which may explain part of the limited competencies of the oocytes seen following IVM. On the other hand, these oocytes represent an indispensable source for deciphering fundamental phenomena. Advances in the systematic analysis of the signals generated in the somatic and germinative compartments of the follicles will help to solve the problems. The prerequisite, however, is that the results are checked against the in-vivo conditions.

## Monozygotic twins and multiples

### Facultative and obligatory polyembryony in mammals

In mammals, sexual reproduction is the rule. In contrast to monotocous/uniparous species with singleton pregnancies, polytocous/multiparous animals normally produce more than one progeny per gestation which might be achieved by multiple ovulations with fertilization of the resulting oocytes or by polyembryony. The latter can be classified as a reproductive strategy in which sexual reproduction is combined with asexual splitting of the fertilization product: During this natural cloning process one fertilized oocyte gives rise to more than one individual. In most mammalian species, regardless of whether uni- or multiparous, this natural process occurs only occasionally (facultative polyembryony), resulting in monozygotic (MZ) twins or multiples, such as in humans (Bulmer, 1970), horses (Meadows et al., 1995; Govaere et al., 2009), cattle (Hancock, 1954; Johansson et al., 1974; Silva del Río et al., 2006; Rogberg Muñoz et al., 2020), pigs (Ashworth et al., 1998; Bjerre et al., 2009), and dogs (Joonè et al., 2016). In contrast, polyembryony seems to be obligatory at least in two of the living armadillo species (Kölliker, 1876; Fernandez, 1909, 1915; Prodöhl et al., 1996; Loughry et al., 1998; Enders, 2002).

Presumably due to the high dizygotic (DZ) twinning frequency in sheep and goats, scientific studies on natural MZ twins are missing in these species and only anecdotal reports are available. However, from observations during preimplantation development in vivo (Assheton, 1898; Rowson and Moor, 1964; Meinecke-Tillmann, 1993) and from the occurrence of conjoined twins (Dennis, 1975; Ahmad et al., 2020) it can be concluded that facultative polyembryony also occurs in small ruminants.

Data on naturally occurring MZ twins indicate a low frequency of about 0.2% to 0.33% per calving in cattle (Johansson et al., 1974; Silva del Río et al., 2006) which corresponds well with the reported frequency of 0.4% per birth in humans (Steinman, 2001).

### Historical aspects

In the 19th century the concept was developed that MZ twins are derived from a single fertilized egg or oocyte. Despite many advances in the field of embryology, there is still uncertainty about the precise mechanisms in which MZ twins or multiples arise. The common hypotheses had been developed on the basis of retrospective analyses of the fetal membranes. Chorionicity and amnionicity allowed the classification into dichorionic-diamniotic (DC/DA), monochorionic-diamniotic (MC/DA) and monochorionic-monoamniotic twins (MC/MA) and gave some indications on the initiation of MZ twin formation via separation of blastomeres or subdivision/splitting of embryos during early development (Corner, 1955), as it was the case with X-chromosome inactivation studies in human embryos (Chitnis et al., 1999).

In contrast, López-Moratalla and Cerezo (2011) and Herranz (2015, 2014) suggested that all types of MZ twins originate from the constitution of “two zygotes” through one longer fertilization process and the subsequent fusion of membranes rather than from the separation of two compartments of an original embryo. However, at least in human embryos (Asami et al., 2022; Perry et al., 2022), first embryonic genes are already activated after male and female nuclear syngamy in the one-cell stage, hence indicating the beginning of embryonic development.

In vivo, separate development of blastomeres or groups of blastomeres in early cleavage stages was postulated to occur in about one-third of human MZ twins and to result in DC/DA individuals, whereas the most common form of MZ twinning, the MC/DA twins, should originate from two separate ICMs (ICM: inner cell mass, embryoblast) in the blastocyst stage (Corner, 1955; Boklage, 1981; Sadler, 2012).

MC/MA MZ twins are not topic of the present review. They are rare and were assumed to emerge after amnion formation, resulting in the growth of two primitive streaks (Corner, 1955) in about 2% to 4% of the cases (Bulmer, 1970; Derom et al., 1995).

These possible modes of MZ twin formation were adopted for other species including farm and companion animals, too.

### DC/DA monozygotic twins

#### DC/DA monozygotic twins associated with atypical or with assisted hatching

The MZ twinning frequency in humans seems to be higher in assisted reproduction programs than during natural MZ twinning and reaches about 1.2% to 4.9% (Blickstein et al., 1999, 2003; Nakasuji et al., 2014; Scaravelli et al., 2022). Data on domestic animals are scarce: A frequency of 1.6% monozygotic multiples had been reported after transfers of single in vitro produced (IVP) equine blastocysts (Dijkstra et al., 2020).

Traditionally DC/DA MZ twin pregnancies were believed to originate early in development, i.e. before the first differentiation into trophoblast and embryoblast (Corner, 1955). This first possibility of twin formation was suggested because of obstetrical evidence, but it has been questioned by experienced embryologists and reproductive physicians: In association with artificial reproductive technologies (ART) the development of DC/DA MZ twins was not uncommon after blastocyst transfer in humans (Peramo et al., 1999; Costa et al., 2001; Kyono, 2013; Sundaram et al., 2018; Li et al., 2020; Dallagiovanna et al., 2021; Brouillet et al., 2022; Chu et al., 2023). This observation was mainly attributed to a disturbed hatching process in the blastocyst stage, with herniation of trophoblast and some ICM cells (Malter and Cohen, 1989). Accordingly, spontaneous MZ twinning or development of MZ multiples in animals occurred after ET of in vivo collected, cultured, frozen, or in IVP-embryos, such as in horses (McCue et al., 1998; Mancill et al., 2011; Roberts et al., 2015; Dijkstra et al., 2020; Peere et al., 2022), cattle (Moyaert et al., 1982; Kraay et al., 1983; Smith et al., 1991), or mice (Chida, 1990; Yan et al., 2015).

Interestingly, in own experiments on assisted hatching (AH) in commercial ET in cattle (Rüther et al., 2002; Rüther, 2005) 3 sets of MZ twins were born after single transfers of in vivo collected embryos in superovulatory cycles but only in the control group (334 transfers of zona-intact embryos, 150 animals calving) and not in the experimental group (324 transfers of zona-manipulated embryos, 177 animals calving).

In contrast to other studies with small zona openings [human zona: <10-30 μm as summarized by Alteri et al. (2018), Sills et al. (2000) and Liu et al. (2022); bovine zona: 7-15x40 μm or at least 40x40 μm: (Schmoll et al., 2003)], a wide slit of the zona pellucida (~ 120 μm slit) was produced by zona dissection in own experiments in order to avoid a disturbed hatching process and, thus, the development of MZ twins in the experimental group (Rüther et al., 2002; Rüther, 2005).

Whereas significant differences in zona pellucida thickness were recognized between human patients (Schiewe et al., 1995), zona pellucida thickness measured at eight different points of the zona, respectively, was homogenous in cattle but differed highly significantly (P < 0.0001) between the developmental stages (Rüther, 2005). Zona thickness was not associated with pregnancy rates (Rüther, 2005), but transfer success was significantly higher after assisted hatching, particularly when fresh first quality rank or frozen/thawed embryos were transferred [pregnancy rates: Rüther et al. (2002); calves born: Rüther (2005)]. Thus, zona hardening during IVC and too small openings in the zona pellucida seem to be associated with disturbed hatching processes in bovine embryos (Schmoll et al., 2003).

In a new study, blastocyst transfers after IVF and AH are confirmed as risk factors for MZ twinning in humans, whereas intracytoplasmic sperm injection, preimplantation genetic testing, and frozen embryo transfer do not appear to be associated with MZ twinning (Chu et al., 2023).

Another own observation after in vivo collection of small ruminant embryos shed some additional light on a possible origin of DC/DA MZ twins or multiples: In a sheep blastocyst which had been collected for embryonic stem cell isolation on D10 of pregnancy, an atypical hatching process in vivo was observed. The zona-entrapped embryo had initiated incomplete hatching and outgrowth of three strangulated vesicles at different areas via tiny openings in a thinned zona pellucida which had not been lost in time (Meinecke-Tillmann, 1993). Provided that each or at least two of these vesicles contained numerically enough ICM cells to support further embryonic development, DC/DA MZ twins or even multiples are conceivable. In this special case the spreading of ICM cells was observed from two of these vesicles during IVC, i.e. from the zona-entrapped blastocyst and from the largest outgrowth. Thus, independent from AH or other in vitro techniques, an altered hatching process in in-vivo developed embryos might result in DC/DA MZ twins or multiples, although the first differentiation into trophoblast and ICM had already occurred.

This assumption is supported by observations in humans (Van Langendonckt et al., 2000; Konno et al., 2020; Brouillet et al., 2022), horses (Dijkstra et al., 2020) and cattle (Massip et al., 1983), and by attempts to induce MZ twins in cattle via a zona-perforation technique (Skrzyszowska et al., 1997, 1999). Linear apoptosis in the ICM might support the twinning process (Ménézo and Sakkas, 2002).

#### DC/DA monozygotic twins associated with double blastocysts

Based on microsurgical experiments with early cleavage stages in small ruminants (Meinecke-Tillmann and Meinecke, 1984b) and on observations on in vivo collected D7 to D12 embryos in sheep (Meinecke-Tillmann, 1993; D0 = day of estrus), it was possible to add some further evidence for the developmental mechanisms of MZ twinning.

Of special interest was the first hypothesis related to the induction of MZ twins during early cleavage but before reaching the compaction stage. This possibility has been questioned because of the easiness of common embryonic development after chimeric embryo aggregation and on the basis of DC/DA MZ twinning seen after single blastocyst transfers when MC/DA MZ twinning had been expected.

Nonetheless, a double blastocyst within a common zona pellucida had been observed which led to the authors’ question: “Monovular twin bovine blastocysts before hatching? Do identical twins sometimes separate this early …?” [ADRI photo unpublished, in Betteridge (1977, p. 78). Unfortunately, the zona pellucida of this specimen was broken as well as partially inverted at the contact area of the two “blastocysts”, and for the smaller structure the presence of an ICM cannot be verified on the basis of the photo. Therefore, it cannot be excluded that a disturbed hatching process had been the origin of a blastocyst connected to a constricted trophoblastic vesicle, pseudo-blastocyst or small blastocyst. This might have occurred via a minor herniation and the subsequent collapse of the original blastocyst with retraction of the strangulated prolapse during the embryo collection procedure.

However, in own investigations on small ruminants, the presence of two separate blastocysts within an intact single zona pellucida was realized in one in vivo developed specimen after its collection on D6 of pregnancy (Meinecke-Tillmann, 1993). After zona removal and separation of these blastocysts, their IVC with the intention to isolate embryonic stem cells resulted in outgrowths of both ICMs. Although the blastocysts had not been transferred into recipient ewes and therefore no twins were born, the observation of two blastocysts within one zona pellucida refutes the assumption of Herranz (2015, p. 5) that “the splitting and growth of twins within the pellucida has been never observed or live-recorded.” 

Moreover, based on earlier microsurgical experiments in farm animals for the induction of intra- and interspecies chimeras in sheep and goats (precisely intergeneric chimeras), some further observations with regard to the possible mechanism of twinning were made (Meinecke-Tillmann and Meinecke, 1984b): When sheep blastomeres of developmentally asynchronous early cleavage stages were combined within a common zona pellucida - in this case a single blastomere of the 4-cell stage (1/4 embryo) with two blastomeres of the 8-cell stage (2/8 embryo) representing together 1/2 embryo - the developing cells formed a single composite blastocyst in 21% of the aggregates after transfer into an intermediate recipient. However, in several cases (39%) the regulation failed, and the parts of the original embryos remained separated and formed two small blastocysts or only one blastocyst without further cleavage of the other component(s) within their common host zona pellucida. The original embryos had been collected on D2 (4-cell stage; D0 = day of estrus) and D3 of pregnancy (8-cell stage), and therefore, the chronological difference between the aggregated blastomeres was about 24 h. A double-zona technique was used to prevent wastage of cells through the slit in the host zona (a small zona pellucida from porcine slaughterhouse material was used as blastomere host, whereas a larger pig zona served as a clamp in order to firmly close and cover the slit in the first one and to prevent blastomere loss; Meinecke-Tillmann and Meinecke, 1984a, b). After blastocyst transfer to the final recipients, some of the half- as well as quarter-embryo derived blastocysts were able to develop into lambs (11/28; 39.3%). On the basis of these results it was suggested that an asynchronous cleavage of the first blastomeres in non-manipulated embryos might lead to the formation of two separate blastocysts within a single zona pellucida, and finally to the development of DC/DA twins (Meinecke-Tillmann and Meinecke, 1984b). Nonetheless one limitation of the observation has to be mentioned retrospectively: Blastomeres of the original 4- and 8-cell embryos had been separated mechanically after a short treatment with Ca- and Mg-free medium. This might have influenced the aggregation readiness, although the blastomeres had been carefully washed after the separation procedure.

As a cause of a naturally occurring blastomere asynchrony, intra- or extrafollicular aging of oocytes had been discussed which might interfere with the quality of the developing embryo (Meinecke-Tillmann and Meinecke, 1984b), whereas superovulation, particularly in combination with ovulation induction, might induce precocious ovulation and extrafollicular aging of oocytes when timed artificial insemination takes place.

Other studies on the aggregation of asynchronous blastomeres also demonstrated difficulties with the regulation of a common embryonic development [rhesus monkeys: Schramm and Paprocki (2004)]. Already Mintz (1965) advised against asynchronous blastomere combination in order to avoid such “parabiotic embryos” which had sporadically been observed by Stern and Wilson (1972) in mice. In this context it should be kept in mind that differences between species exist, particularly with regard to regulative and regenerative competency and capacities of the early embryo [e.g., Kohri et al. (2019)]. Furthermore, differences in adhesiveness of blastomeres might play a role in the possible formation of aggregates (Kimber et al., 1982).

Asynchronous blastomere cleavage after IVF was observed in humans and mice and was associated with lower ICM quality and higher abortion rates (Mashiko et al., 2022). A safe cryopreservation of “synchronous” as well as “asynchronous” embryos (cryopreserved 2 or 3 days after oocyte aspiration) was possible in women, but, unfortunately, detailed information is missing, although twins had been born (Wiener-Megnazi et al., 2014).

Furthermore, Bomsel-Helmreich (1974) and Bomsel-Helmreich and Papiernik-Berkhauer (1976) reported MZ twin blastocysts within the same zona pellucida after delayed ovulation in rabbits. Delayed ovulation results in intrafollicular aging of the oocytes. Their minor quality was associated with high embryonic mortality and chromosomal anomalies as well as the occurrence of monozygotic twins. On the basis of cytogenetic investigations the authors hypothesized that twins of the same sex must have their origin in the 2-blastomere-stage, i.e. at the same time when mixoploids arise.

### MC/DA monozygotic twins

In MC/DA MZ twins other mechanisms must be active than in those with DC/DA membranes. It was suggested that two inner cell masses might occur within a single zona-enclosed blastocyst via ICM-splitting or -duplication. Accordingly, three in vivo developed normal sized sheep blastocysts, each with two separate inner cell masses at opposite poles of the zona-enclosed preimplantation embryos were collected from different superovulated ewes on D7 of pregnancy (Meinecke-Tillmann, 1993).

The development of two separate ICMs might be induced by disturbances of blastocoel formation or purely mechanically. The latter had been suggested on the basis of blastocyst collapse and re-expansion in human embryos (Payne et al., 2007; Mio and Maeda, 2008). Factors controlling cavity formation and the positioning of the eccentric fluid-accumulation are not completely understood. In mice, a hydraulic flux fractures cell-cell contact in a network of microlumina which empty themselves into larger ones until a single cavity results. Thus, blastocoel formation in mice and presumably in other species such as cattle or humans depends on functional ion transport through a polarized epithelium as well as hydraulic and osmotic phenomena as indicated by Dumortier et al. (2019) and Le Verge-Serandour and Turlier (2022). Usually, trophectoderm cells flatten under increased pressure which is believed to ensure that there are no asymmetric divisions or additional ICM cells formed after the blastocyst cavity reaches a certain size (Chan et al., 2019). This stresses the importance of mechanical influences during early development.

The formation of multiple blastocoelic cavities was considered to be abnormal (Alikani et al., 2000). It is unknown if such a situation can result in an altered positioning of prospective ICM cells. The development of strings and bridges between ICM and trophoblast during human blastocyst growth [summarized by Hardarson et al. (2012)] might influence blastocyst quality and might further be involved in the shaping of the ICM and therefore in the process of twinning. Mechanical influences on blastocyst shaping are gaining increasing interest (Özgüç and Maître, 2020; Firmin and Maître, 2021), and their actions should also be considered during the peri-implantation period.

Monozygotic double ICMs have been reported in mouse blastocysts, too. They were recognized after in vivo or in vitro fertilization and subsequent in vitro culture from the 2-cell up to the blastocyst stage (Chida, 1990), or after in vivo fertilization and subsequent in vitro culture of blastocysts up to the egg cylinder stage (Hsu and Gonda, 1980). In the latter example the double ICMs were induced purely mechanically. In this context it should be kept in mind that in vitro situations are prone for artifacts, although it cannot be excluded that a disturbed intrauterine embryo-orientation might also be involved in monozygotic twinning processes in vivo.

Furthermore, Otsuki et al. (2016) recommended the exclusion of in vitro produced blastocysts that contain decompacting ICMs from transfer in order to avoid monozygotic MC/DA twinning via a doubling of the ICM. On the basis of time-lapse photography, it was possible to ascribe one case of human MC/DA twins to the transfer of a blastocyst with a decompacted ICM of at least eight cells (Otsuki et al., 2016). Inner cell mass morphology is associated with embryo quality and ET success (Subira et al., 2016; Ai et al., 2021; Yaacobi-Artzi et al., 2022), and the appearance of blastocysts with “loosely arranged” inner cell mass cells and tightly packed trophectoderm (Shi et al., 2021), or with tightly packed trophectoderm (Ge et al., 2022) is related to the development of human monozygotic twins. Even though an assessment of the latter studies is difficult since relevant data is missing, both investigations indicate that the fate of the ICM in in vitro produced preimplantation blastocysts remains labile, thus making a reorganization and development into singletons or twins possible. Interestingly, in humans the extremely rare event of familial MZ twinning was reported which seems to be associated with cell junction-signaling pathways (Liu et al., 2018).

In a further human blastocyst, the presence of two ICMs was recognized, both differing in the stage of development (Noli et al., 2015a). This indicates a certain autonomy of a group of pluripotent inner cells with the growth of a second ICM rather than the splitting of the first one. In this context it is known that the trophectoderm of human (Paepe et al., 2013) and cattle embryos (Berg et al., 2011) can under special conditions still contribute to the ICM. Despite these observations, Herranz (2014) postulated that all human MZ twins start as dichorionic twins and may become monochorionic via trophectoderm fusion.

Interestingly, even a monochorionic triamniotic pregnancy resulted after transfer of an 8-shaped hatching blastocyst with two ICM structures (Sutherland et al., 2019). In contrast to the above mentioned publications, Gu et al. (2018) stated that an ICM incarceration in 8-shaped blastocysts does not increase the incidence of MZ twins in humans. This might indicate that the duration of an ICM incarceration is a relevant factor.

It should be emphasized that MZ human twins carry a robust DNA methylation signature in adult somatic tissues at genes involved in processes including cell adhesion, WNT signaling and cell fate (Van Dongen et al., 2021). As indicated above, cell adhesion might also play a role in the development of either DC/DA MZ twins derived from early cleavage stages or of MC/DA MZ twins derived from blastocysts with double ICMs. The Wnt/ß-catenin pathway plays a role during early development and maintenance of pluripotency (Denicol et al., 2013; Sidrat et al., 2020; Kinoshita et al., 2021; Liu et al., 2021; Xiao et al., 2021).

A human blastocyst with two ICMs, atypically resulting in DC/DA MZ twin embryos, was described by Meintjes et al. (2001). Although the authors stated that “[...] this case of monozygotic twinning can not be explained by in vitro zona alteration” Meintjes et al. (2001, p. S173), a disturbed hatching process with sequestration of a trophoblastic vesicle containing ICM cells is the most obvious cause for this atypical dichorionicity.

In addtion to the above mentioned pre-hatching sheep blastocysts with two ICMs, one in vivo developed elongating ovine blastocyst with two embryonic discs was collected from a superovulated donor ewe on D11 of pregnancy (Meinecke-Tillmann, 1993). Such specimens which theoretically might result in MC/DA monozygotic twins were already described by Assheton (1898) in an elongating sheep embryo which can be estimated to be about 10 to 11 days old [Bindon, 1971; Meinecke-Tillmann, 1993; although an age of D7 had been published by Assheton (1898)], and by Rowson and Moor (1964) in four sheep embryos collected between D6/7 and D14 of gestation. In our D11 embryo it cannot be completely ascertained whether the twin embryonic discs originated from ICM duplication or fission, or from a fusion of two zona-free blastocysts. Regarding the position of the embryonic discs and the absence of any trophoblastic strictures in the D11 conceptus, a previous ICM duplication within a single blastocyst is most likely, and MZ twins would result.

### Blastocyst fusion and twinning

In the case of blastocyst fusion MC/DA DZ twins instead of MZ twins would be expected, as long as the ICMs remain separated. This event results in a temporary primary chimerism and must be extremely rare in in-vivo grown developmental stages since pre-hatching embryos are preferred for commercial ET in ruminants. Even after the transfer of two embryos, fusion cannot be expected because the blastocysts are usually transferred into different uterine horns, avoiding a close contact to each other. Likewise we never observed fusion of hatching or early post-hatching blastocyst stages during IVC of ruminant embryos.

The readiness for aggregation and development of firm interconnections might be dependent on the species, the physiological time of embryo-attachment to the endometrium, and the type of implantation. Mouse and human blastocysts show very little expansion before early implantation soon after zona shedding. In contrast, implantation in ungulate species is superficial and delayed. Therefore, an early fixation of the conceptus would hamper further development. Accordingly, we recognized conjoined blastocysts which were flushed from the uteri of three superovulated ewes not before D10/D11 of pregnancy. In two pairs of these still spherical D11-blastocysts obtained from two of these ewes, respectively, a large superficial attachment zone between the trophoblasts was present, including about one sixth of their surface and resulting in a local flattening of the connected spheres. Both blastocyst pairs could be pulled apart without any tissue loss with the help of two microtools. In contrast, a separation without severe tissue damage was not possible in a group of five spherical D10-blastocysts recovered from the third ewe. They were conjoined via cell projections at punctuated trophectodermal contact areas. The central one was interconnected with every other blastocyst, whereas the lateral ones showed firm connections with the central one as well as with their direct neighbor. Confluence of blastocoels was absent in every entity, and each of the involved blastocysts contained a normal sized ICM. It is realistic to assume that both of the more loosely interconnected and otherwise normal blastocyst pairs might have resulted in physiological pregnancies, although the growth into a filamentous conceptus and the orientation of the embryo in relation to the uterine luminal epithelial layer might have been hampered if the conjunction between the two blastocysts would have stabilized.

The group of five firmly interconnected zona-free blastocysts resembled the above mentioned embryo with atypical hatching in different small areas of the thinned zona pellucida (see 3.3.1). Possibly such a zona entrapment is not permanent as long as the embryo is viable and proceeds in development. However, each of these five equal sized spheres possessed a normally developed ICM and trophoblast which suggests secondary fusions of blastocysts during the post-hatching period. It is difficult to speculate on a possible further development of these firmly interconnected zona-free structures. Trophoblast fusion might have a negative influence on embryo attachment, implantation and placentation. Furthermore, permanent interconnection might disturb embryo spacing and result in a crowding-effect in one uterine horn with negative consequences for embryonic survival.

Only few reports on early blastocyst fusion are available in the international literature. In mice, fusions during in vitro culture were induced with fusogenic viruses (Tarkowski and Wojewodzka, 1982: inactivated Sendai virus) or electrofusion (Ozdzeński et al., 1997; Tarkowski et al., 2005). In this context it should be noted that the electrofusion of zona-free mouse blastocysts allowed the development of common trophoblastic vesicles containing either one aggregated or two separate ICMs which was dependent on the prior orientation of the inner cell masses (Tarkowski et al., 2005). Thus, fusion of blastocysts might be a mechanism of twinning but - as mentioned above - of MC/DA DZ twins. This rare event had also been recognized in group-cultured human blastocysts after laser dissection of the zona pellucida (Schiewe et al., 2015), and spontaneous trophectoderm amalgamation was observed twice between two hatching blastocysts, respectively. Spontaneous fusions of group-cultured human blastocysts were further described by Swain (2021). Thus, it would be advantageous to avoid group-culture systems which allow a close contact between the individual embryos.

Moreover, fusion of blastomeres occurred after freezing and thawing of early human cleavage stages through membrane destabilization induced by cryoprotectants (Balakier et al., 2000).

Interestingly, the development of human MC/DA MZ twin pregnancies had also been reported after zona-free blastocyst transfer (Frankfurter et al., 2004) but, unfortunately, the data presented are incomplete and do not allow a final interpretation. Although Frankfurter et al. (2004) stated that monozygotic twins resulted from zona-free blastocysts, they disregarded the possibility of blastocyst fusion which may result in temporary or permanent chimerism and DZ twinning. Data are missing which are related to the number of blastocysts that had been transferred to the individual women getting pregnant with twins (presumably two blastocysts since “normal” DZ multiples were also reported). Furthermore, the possible monozygosity had only been determined on the basis of ultrasonic scans during early pregnancy, whereas monozygosity at birth or pathology was only confirmed “when possible”, and the report of appropriate criteria is also missing.

From conjoined oocytes which have occasionally been seen after oocyte retrieval in humans and which can result in successful pregnancies (Magdi, 2020; Wang et al., 2022), only singletons, DZ twins or chimeras would be expected after fertilization, unless other mechanisms which have been reported above contribute to twin formation

### Artificially induced monozygotic twins or multiples

#### Splitting of early preimplantation embryos

As could be demonstrated, the early mammalian embryo exhibits a remarkable plasticity, and its cells are able to respond rapidly to damaging conditions. Interest in the tremendous regulative capacities of early embryos led to investigations including experimental blastomere isolation, blastomere isolation and aggregation, embryo halving, quartering, or separation into eights, and trials related to the artificial induction of monozygotic twins or multiples. This has been accomplished in a variety of species, such as humans (experiments not further than up to preimplantation stages: Hall et al., 1993; Van de Velde et al., 2008; Illmensee et al., 2010; Noli et al., 2015b; Omidi et al., 2020), monkeys (Mitalipov et al., 2002), horses (Allen and Pashen, 1984; Skidmore et al., 1989), cattle (Willadsen et al., 1981; Willadsen and Polge, 1981; Ozil et al., 1982; Voelkel et al., 1985; Warfield et al., 1987; Johnson et al., 1995; Rho et al., 1998; Skrzyszowska et al., 1999; Hashiyada, 2017), sheep (Trounson and Moore, 1974; Meinecke-Tillmann et al., 1979; Willadsen, 1979, 1980, 1981; Meinecke-Tillmann, 1980, 1993; Meinecke-Tillmann and Meinecke, 1981, 1983b, 1987), goats (Meinecke-Tillmann and Meinecke, 1983a, 1987; Tsunoda et al., 1985; Udy, 1987; Nowshari and Holtz, 1993), pigs (Nagashima et al., 1989; Reichelt and Niemann, 1994; Dang-Nguyen et al., 2011), rabbits (Yang and Foote, 1987), rats (Matsumoto et al., 1989), and mice (Mullen, 1971; Moustafa and Hahn, 1978; Gärtner and Baunack, 1981; Tsunoda and McLaren, 1983; Nagashima et al., 1984; Tsunoda et al., 1987; Carstea et al., 2007; Katayama et al., 2010; Tarkowski et al., 2010; Zhang et al., 2018; Krawczyk et al., 2021; Maemura et al., 2021). Success rates differed according to species, manipulated developmental stages and manual skills of the operator.

In farm animals the first artificially induced monozygotic twins were reported in sheep after separation of blastomeres of very early cleavage stages (Willadsen, 1979), or after bisection (splitting) of morulae and blastocysts (Meinecke-Tillmann, 1980; Meinecke-Tillmann and Meinecke, 1981).

Although, at least in farm animals, demi-embryos are nearly as suitable for the establishment of pregnancies as intact embryos, the developmental potential of single blastomeres of the 4- or 8-cell stage is more limited than that of the 2-cell stage (Willadsen, 1981; Krawczyk et al., 2021).

Whereas simple division of preimplantation embryos has occasionally been successful for producing up to monozygotic quadruplets, serial splitting was introduced in the hope to create higher order multiples, and in vitro trials on serial splitting of mouse or bovine cleavage stages were reported (Illmensee et al., 2006; Silvestri et al., 2022). In this context it might have been overlooked that, despite of the impressive regulative capacities, a developmental clock regulating polarization and blastocyst formation is present in preimplantation embryos. This was already indicated, for example, by Tarkowski (1959) and Tarkowski and Wroblewska (1967), and demonstrated by other authors (Pratt et al., 1981; Johnson et al., 1984; Dean and Rossant, 1984; Prather and First, 1986; Modliński et al., 2002; Lorthongpanich et al., 2012; Noli et al., 2015b; Zhu et al., 2020; Maemura et al., 2021). Until now, this developmental clock prevents the multiplication of animals via serial splitting of cleavage stages.

#### Monozygotic multiples via chimeric cloning

The only theoretical possibility to produce monozygotic multiples of higher order is “chimeric cloning”. This can be performed either via blastomere complementation (sheep quintuplets: Fehilly and Willadsen, 1986; sheep triplets: Meinecke-Tillmann, 1993), or via stem cell complementation. In mice, embryonic (ESC), parthenogenetic or induced pluripotent stem cells (iPSC) were successfully combined with blastomeres, tetraploid blastomeres, tetraploid blastocysts, or trophoblastic vesicles (Nagy et al., 1990, 1993; Modliński et al., 2004; Huang et al., 2008; Boland et al., 2009, 2012; Chen et al., 2009; Zhao et al., 2009, 2010; Sumiyama et al., 2018), whereas in cattle only low-grade chimeric progeny resulted after aggregation of ESC-like cells with presumably tetraploid embryos (Iwasaki et al., 2000). Unfortunately, in contrast to mice, a high grade of mosaicism between diploid and tetraploid cells occurred after electrofusion of blastomeres in cattle (Curnow et al., 2000).

Our experimental approaches with regard to embryonic stem cells, for example in sheep and goats, dated back to 1991 (Meinecke-Tillmann and Meinecke, 1991, 1996; Meinecke-Tillmann, 1993), but trials to establish ESC-lines from farm animals posed technical problems. Nowadays, some breakthroughs have also been achieved in farm animals [see reviews of Navarro et al. (2019); Kim and Roh (2021); Kumar et al. (2021); Aguila et al. (2022)]. This might enhance the technique of chimeric cloning.

Meanwhile, the suitability of trophoblastic vesicles with ICM-exchange (i.e. the induction of a temporary chimerism) was demonstrated as a promising approach for endangered species conservation, although until now, in contrast to earlier experiments with asynchronous blastomere aggregation (Meinecke-Tillmann and Meinecke, 1984a) only intraspecies progeny has been born (Loi et al., 2018). Before, successful ICM-replacement was performed between differing mouse stains (Bi et al., 2003; Zheng et al., 2005) and with ICMs derived from bovine nuclear transfer embryos and trophoblastic vesicles of bovine IVP-embryos (Murakami et al., 2006). However, it might become possible to generate gametes from iPSC of endangered animals that can be used to create IVP-blastocysts and to transfer their isolated ICMs into trophoblastic vesicles derived from a suitable species which carries the interspecies chimeric blastocysts to term (Saragusty et al., 2020). A similar approach is conceivable with ICMs from IVP-blastocysts after interspecies cloning with somatic cells or iPSC of an endangered species and oocytes from another closely related and compatible but unthreatened species. Unfortunately, in these cases mtDNA of a foreign species is undesirably transmitted.

Since ectogenesis is far from reality it is necessary to find suitable and common species that can serve as foster mothers for the interspecies approach in animals that are on the brink of extinction. In the context of endangered animals it should not be forgotten that non-mammalian species deserve attention, too (Lipke et al., 2009b, a; Strand et al., 2020; Bolton et al., 2022).

### The zona pellucida in micromanipulated embryos

The zona pellucida (Moros-Nicolás et al., 2021) protects pre-compaction embryos from lysis, immobilization, aggregation, disaggregation or loss of blastomeres, and also from contact with immune cells or infectious material (Modliński, 1970; Nichols and Gardner, 1989; Ueno et al., 2007). Additionally, the zona is necessary for the establishment of a special microenvironment. Furthermore, it eases handling during ET procedures. Thus, for the protection of zona-injured cleavage stages before reaching the compacted morula stage, different methods were tested or established such as encapsulation in agar (Willadsen, 1979; Willadsen et al., 1981; Tsunoda and McLaren, 1983), agarose (Blakewood et al., 1989), or agar/agarose cylinders (Meinecke-Tillmann, 1993), sodium alginate (Adaniya et al., 1987; Cosby and Dukelow, 1990; Hall et al., 1993; Watanabe et al., 1995; Yániz et al., 2002), or poly-l-lysine (Krentz et al., 1993), protection with a double zona pellucida (Meinecke-Tillmann and Meinecke, 1984a, b; Meinecke-Tillmann, 1993), gelatin embedding (Warfield et al., 1987), or zona substitution with special agarose capsules (Nagatomo et al., 2017) or sodium hyaluronate gel (Song et al., 2022). In the case of encapsulation with non-degradable material the embryos had to be freed in the late morula or early blastocyst stage.

Before embedding in foreign material, manipulated embryos / blastomeres normally were surrounded with a host zona pellucida. These host zonae were usually taken from unfertilized / degenerate oocytes or embryos that were recovered during embryo collection in vivo or during in vitro culture, or from oocytes collected from abattoir material. In our own studies fresh zonae pellucidae derived from oocytes of prepubertal pigs were preferred (Meinecke-Tillmann and Meinecke, 1983b, 1984a, b, 1987; Meinecke-Tillmann, 1993) because of their smaller size which made them more suitable for splitted or otherwise treated embryos, and because of the absence of pre- and post-fertilization hardening which might unfavorably interfere with blastocyst hatching [zona hardening: see Coy et al. (2008)]. The double zona technique allowed, if desired, the direct transfer into final recipients. Nevertheless, it cannot be excluded that the mechanics during hatching might have been of negative influence on pregnancy and embryo survival rates, since these were not as high as after agar/agarose embedding/removal and ET to intermediate recipients (Meinecke-Tillmann, 1993). On the other hand it has to be taken into account that embedding in agar/agarose cylinders allowed a preselection of embryos and therefore higher success rates after ET.

Hygienic precautions had been taken into account since the pig zonae were derived from slaughterhouse material. Therefore, they were carefully denuded, evacuated and washed in order to remove possible contaminants. In contrast to former observations (Moore et al., 1969), a foreign zona pellucida does not negatively influence the pre-hatching development.

The necessity of zona-envelopment had been questioned by Feltrin et al. (2014) since low embryonic survival rates were observed after oviductal transfers of both zona-free and zona-enclosed cloned goat D1- to D2-embryos on D30 of pregnancy (presence of an embryo proper: 5.6% vs. 5.8%; with heartbeat: 0.7% vs. 0.6%). In this context it should be kept in mind that cloned embryos are a suboptimal approach to investigate the problem of a missing or injured zona pellucida. Even when advanced IVC- methods are used (Park et al., 2015), the enclosing in a host zona pellucida might still be of interest, depending on the species, the preimplantation stage, and the type of manipulation. In general, however, zona-free embryos develop in vitro at a similar rate to blastocysts as zona-enclosed embryos (Lagutina et al., 2007), and zona-hardening in vitro and its consequences are undesirable (Madani et al., 2022). Thus, the necessity of the presence of the zona pellucida in modern embryo culture systems (Hashiyada, 2017; Fan et al., 2022) has to be questioned. Nonetheless, further investigations are required, since it had been shown that zona removal affects, for instance, blastomere conformation (Katayama et al., 2010) and gene expression as well as pre- and postimplantation development in mice (Fan et al., 2022), although influences might again be species specific (cattle: Velásquez et al., 2013).

Stickiness of zona-free blastocysts can be overcome by proper handling of the embryos, and, thus, ET of micromanipulated zona-free post-compaction stages did not lower pregnancy rates in comparison to the control group (Warfield et al., 1987).

### Maternal effects

Embryo technologies per se (Betteridge, 1977, 1981; Biggers, 2012; Hansen, 2020a, b) and artificial production of monozygotic twins can be involved in breeding programs or in comparative experiments (e.g. Wassmuth and Meinecke-Tillmann, 1980; Biggers, 1986; Kippax et al., 1991; Weppert, 2006; Hashiyada, 2017; Casser et al., 2019a; Mueller and Van Eenennaam, 2022).

One aspect that shall be discussed here in some more detail is the influence of the maternal genotype or phenotype on the phenotype of the offspring (Wolf and Wade, 2009) since birth weight is a relevant factor for progeny survival and health.

Studies on maternal effects initially involved ET between large and small breeds or reciprocal exchange of embryos between strains or breeds in different species, including sheep (Hunter, 1956; Karihaloo and Combs, 1971; Meinecke-Tillmann and Wassmuth, 1977; Hinkelman et al., 1979; Anderson et al., 1981; Naqvi et al., 2006; Emsen et al., 2012; Sharma et al., 2012; Oliver et al., 2015), pigs (Smidt et al., 1966; Steinbach et al., 1967), and mice (McLaren and Michie, 1958; Brumby, 1960; Cowley et al., 1989) which were later followed by experiments on horses (Allen and Pashen, 1984; Tischner, 1987; Tischner and Klimczak, 1989; Allen et al., 2002; Peugnet et al., 2017) and cattle (Guilbault et al., 1990; Gregory and Maurer, 1991). They were mainly performed to investigate the impact of a more “comfortable” uterine environment in comparison to a restricted one on the pre- and postnatal development of body weight and size of the fetus/newborn, and on the duration of pregnancy. In contrast, studies related to birth weight in cattle primarily concentrated on the effects of artificial reproductive techniques (King et al., 1985; Lopes et al., 2022).

Although Emsen et al. (2012) observed no recipient breed effect in sheep, significant influences were recognized in most of the investigations and reflected the regulatory effects of the uterine environment on either birth weight or weaning weight of lambs.

Depending on the breed, larger/heavier genotype lambs were smaller/lighter when born to smaller/lighter genotype dams (e.g., Hunter, 1956; Karihaloo and Combs, 1971; Meinecke-Tillmann and Wassmuth, 1977; Sharma et al., 2012). However, contradicting results had been obtained with regard to birth weight or body dimensions of small genotype lambs that were born to larger embryo recipients. Whereas significant differences were seen in comparison to the controls when smaller/lighter genotype lambs were delivered by larger/heavier genotype foster mothers (e.g., Karihaloo and Combs, 1971; Meinecke-Tillmann and Wassmuth, 1977), this was not the case in another study (Sharma et al., 2012). Similar to sheep, birth weight of calves was higher in Ayrshire dams bearing Limousin fetuses than in those bearing Ayrshire fetuses (Guilbault et al., 1990) which was compensated within 5 months of postnatal development. Dependent on the breed, differences between control lambs and lambs that were delivered by foster mothers were compensated between 14 d and 8 weeks of age (Meinecke-Tillmann and Wassmuth, 1977) or after 8 months (Hunter, 1956), whereas in horse fillies (Pony and Thoroughbred), differences in birth weight as well as in other parameters resulting from transfer to host mothers were still obvious at 3 years of age (Allen et al., 2004). Similar to sheep, compensation of the influence of the intrauterine environment on prenatal development of piglets already took place during the first 4 to 5 weeks of life (Smidt et al., 1966).

Since postnatal growth of progeny is influenced by milk production of the individual dams, and since milk production itself depends on the breed, birth type and sex of the newborn (Guilbault et al., 1990; Hinde et al., 2014; Abecia and Palacios, 2018) as well as the breed of the dam, it was sought to objectify the results obtained during the time period from birth until weaning in a few studies. This was achieved by artificial rearing of the lambs (Meinecke-Tillmann and Wassmuth, 1977), or by uniform foster mothers and a defined number of pups per female in mice (Cowley et al., 1989).

Pregnancy duration was longer in dams bearing progeny with higher birth weight (e.g. sheep: Meinecke-Tillmann and Wassmuth, 1977; cattle: Guilbault et al., 1990). In this context, it should be kept in mind that in contrast to several other mammals the strategy of timing of parturition in sheep depends solely on the fetal hypothalamic-pituitary-adrenal axis (Liggins, 1974; Rokas et al., 2020). Thus, in other species differing results might be expected.

In order to specify effects of the maternal environment, matching MZ demi-embryos had been transferred into a single or into two different foster mothers (sheep: Meinecke-Tillmann, 1980, 1984; horse: Allen and Pashen, 1984; Allen, 2005) since a mere transfer of embryos between large and small breeds left questions open. Although only small groups of monozygotic twins were available in these studies, results indicated that lambs derived from demi-embryo pairs which had been transferred to two ewes of similar size were more similar to each other than after transfer to different uterine horns of a single mother. In the latter case, restricted growth of the smaller MZ twin was compensated within three month after delivery. Pregnancy duration was identical in MZ twin pairs born from two different but similar sized ewes (same day, difference of only few hours; Meinecke-Tillmann, 1984). In contrast, MZ fillies were born 23 d apart to two mares of different body size and showed a marked dissimilarity in birth weight which was not completely compensated in later life (Allen and Pashen, 1984). In another group of MZ horse twins the growth rate was either enhanced or curbed depending on the genetic background of the foals in the first 6 months post partum, and a minor effect of the uterine environment persisted in the mature animals (Allen et al., 2004).

Since in sheep the size of each twin at birth might largely be determined during early gestation (Hancock et al., 2012), further investigations on monozygotic twins carried by a single or by different foster mothers are recommendable. Ultrasonography is suitable to follow the intrauterine development noninvasively (Meinecke-Tillmann and Meinecke, 2007; Elmetwally et al., 2016a, b; Meinecke-Tillmann, 2017). In this context it would be of particular interest that matching demi-embryos can successively be carried by the same or by different dams, such as in different seasons or under other differing environmental circumstances. With regard to animal behavior, reciprocal embryo transfers between anxious or non-anxious individuals or between breeds of different temperament would offer new perspectives, particularly when MZ twins would be investigated.

Monozygotic sheep and cattle twins of different age were already born after one demi-embryo had been transferred directly after collection and micromanipulation, whereas the other demi-embryo had been stored at -196°C for several weeks (Willadsen, 1980; Seike et al., 1991). This approach also allowed successful pregnancies in goats carrying their MZ twin conceptus (Oppenheim et al., 2000). Similar to goats, a syngeneic pregnancy was also successful in horses, although in cloned individuals (Galli et al., 2003). The MZ twin model has further been used for investigating the embryo-maternal dialogue in cattle (Klein et al., 2006).

Artificial monozygotic twinning would be helpful for the differentiation between true maternal effects [see Wolf and Wade (2009)] and other factors such as maternal inheritance or genomic imprinting. That maternal effects can override any genetic control was impressively demonstrated with regard to the development of endometrial cups in a mare and a jenny donkey, each pregnant from one of two matching MZ mule embryo halves (Allen et al., 1993).

Blastocysts/conceptus derived from demi-embryos might differ from each other, for instance since dissimilarities between cleavage products [Casser et al. (2019b), discussed by Denker (2020)] or universal epigenetic inter-individual dissimilarities (Planterose Jiménez et al., 2021) may exist, the maternal environment (Ollikainen et al., 2010) or assisted reproductive technologies may influence DNA methylation/gene expression (Urrego et al., 2014; Velásquez et al., 2016, 2017; Barberet et al., 2022; Håberg et al., 2022), or postzygotic mutations may occur in somatic cells as well as germ cells (Jonsson et al., 2021).

In this context, the transfer of MZ demi-embryos into MZ twin mothers might be of special interest for further investigations.

### Conclusion

The mammalian preimplantation embryo is equipped with amazing regulative capacities which seem to be both species and stage specific. This reproductive strategy allows the compensation of a loss of defective blastomeres even at two-cell stages or the regulation of ploidy without endangering the whole pregnancy, especially in uniparous species with their long generation intervals. Therefore, the cellular and embryological basis for twinning might vary between species. The high regulative capacity also allows the artificial splitting of preimplantation embryos in order to increase the number of transferable embryos per donor animal.

Regarding the process of spontaneous monozygotic twinning in association with natural pregnancies as well as artificial reproductive technologies, hypotheses on the development of dichorionic/diamniotic and monochorionic/diamniotic twins are discussed on the basis of selected international publications and of own observations of in vivo developed or in vitro manipulated embryos in small ruminants. Contrary to the general view that twin blastocysts cannot occur within a common zona pellucida, we describe such cases in native and in manipulated embryos. Furthermore, possible mono- or dizygotic twinning related to blastocysts with double ICMs or to fused blastocysts are reported. MZ twins offer the possibility to perform comparative studies in which one twin serves as the control. This is of special interest with regard to maternal influences on the developing conceptus.
